# Novel Methodology for Football Rebound Test Method

**DOI:** 10.3390/s20061688

**Published:** 2020-03-18

**Authors:** Enrique Colino, Lis Corral-Gómez, David Rodríguez-Rosa, Sergio Juárez-Pérez, Jorge García-Unanue, Antonio González-Rodríguez, Javier Sánchez-Sánchez, Jose Luis Felipe, Leonor Gallardo, Fernando Jose Castillo-García

**Affiliations:** 1 IGOID Research Group, University of Castilla-La Mancha, 45071 Toledo, Spain; Enrique.Colino@uclm.es (E.C.); Jorge.GarciaUnanue@uclm.es (J.G.-U.); Leonor.Gallardo@uclm.es (L.G.); 2 School of Industrial and Aerospace Engineering, University of Castilla-La Mancha, 45071 Toledo, Spain; lis.corral@uclm.es (L.C.-G.); David.RRosa@uclm.es (D.R.-R.); Sergio.Juarez@uclm.es (S.J.-P.); Antonio.Gonzalez@uclm.es (A.G.-R.); 3 School of Sport Sciences, European University of Madrid, 28670 Madrid, Spain; javier.sanchez2@universidadeuropea.es (J.S.-S.); joseluis.felipe@universidadeuropea.es (J.L.F.)

**Keywords:** sport surface, ball rebound test, mechatronics, robotics, repeatability

## Abstract

Assessing and keeping control of the mechanical properties of sport surfaces is a relevant task in sports since it enables athletes to train and compete safely and under equal conditions. Currently, different tests are used for assessing athlete- and ball-surface interactions in artificial turf pitches. In order to make these evaluations more agile and accessible for every facility, it is important to develop new apparatus that enable to perform the tests in an easier and quicker way. The existing equipment for determining the vertical ball behavior requires a complex and non-easily transportable device in which the ball must be fixed to the upper part of the frame in a very precise position by means of a magnet. The rebound height is determined by capturing the acoustic signal produced when the ball bounces on the turf. When extended tests are conducted, the time required to evaluate a single field is too high due to the non-valid trials. This work proposes a novel methodology which allows to notoriously decrease the time of testing fields maintaining the repeatability and accuracy of the test method together with a compact device for improving its mobility and transport. Simulations and experiments demonstrates the repeatability and accuracy of the results obtained by the proposed device, which decreases the non-valid trials and notoriously reduces the time for field evaluation.

## 1. Introduction

Artificial turf football pitches are one of the most commonly used sports facilities worldwide. These facilities contribute significantly to the development of football at all levels and all ages, representing a very important resource for sports practice in most societies and contexts [1]. Artificial turf systems offer considerable advantages compared to natural grass pitches since the formers are much less sensitive to frequent use and inclement weather [2,3]. Although first-and-second generation artificial turf had some disadvantages such as distorted behavior of the ball and increased risk of injury [4] with the connected social costs for orthopedic [5] or dental rehabilitations [6], the quality of modern artificial turf systems is such that the governing bodies of football currently accept the use of third-generation artificial turf systems in official competitions. These systems are characterized by long fibers in the carpet (40–65 mm), a relatively low tuft density, and, most of all, the presence of large quantities of infill in comparison to first or second generation products, with usually a sand layer at the base and crumbed rubber at the top [7]. Also, a shockpad layer is often placed underneath the grass carpet [8].

Previous studies have identified the condition of the playing surface as a parameter that affects football players’ safety and performance [2,9,10]. Therefore, to promote football in a responsible way and to safeguard the safety and the integrity of the performance of all athletes, it should be permanently guaranteed that these surfaces comply with appropriate quality standards [8]. To do so, any artificial turf football pitch, new or used, should be submitted periodically to maintenance tasks and evaluation tests to ensure that its surface has been installed correctly and meets those quality standards. Whilst the maintenance tasks would depend on different factors such as hours of use, climate conditions or wear, the evaluation tests will allow to assess anytime the sport functionality of the surface, helping to plan the maintenance activities more effectively and increasing the impact of these actions and the performance and durability of the artificial surface.

The on-site evaluation of artificial turf pitches is developed using mechanical devices that enable properties to be measured directly on the installed surface by performing tests that somehow reproduce ball and athlete interactions with the ground in a real game situation. Although some of these tests might be excessively simplistic when mimicking human and ball movement [11,12,13,14], they still are recognized by most of the international standards and sports federations as being appropriate for the assessment and regulation of sports surfaces, since they represent a practical and reproducible tool for predicting surface behavior during athletic movements. Therefore, mechanical tests are useful because they allow surfaces to be compared and provide evidence whether they meet certain specification standards [15].

Two different organizations have issued regulations seeking to ensure quality of artificial turf football pitches: the European Committee for Standardization (CEN) and the Fédération Internationale de Football Association (FIFA). Thus, the European standard EN 15330-1 [16], issued by the CEN, and the Quality Programme for Football Turf [17], issued by the FIFA, are the two regulations under which the quality of an artificial turf football pitch can be evaluated. While the EN 15330-1 standard is intended to apply to surfaces used for community, educational and recreational sport, FIFA specifications are more focused on professional and elite levels of competition.

Generally, the tests methods stipulated in both the CEN and the FIFA regulations are old and complex, and due to the current development of technology they are certainly becoming out of date and, in some cases, inaccurate. This adversely affects the quality control of artificial turf football pitches as it slows down the data collection processes and increases the margins of error and the evaluation costs. Thus, although some other sports federations faced the task of renewing their test methods several years ago [18], most of the methods currently applicable to artificial turf football pitches have been outdated for a long time. Therefore, to develop simpler test methods that allow a quicker and more accessible evaluation of artificial turf surfaces is a measure that would benefit all parties, from the managing bodies of the facilities to the athletes themselves.

The requirements that installed artificial turf football surfaces must meet to ensure that the facility is suitable for the intended use can be generally divided into two different groups: those affecting athlete-surface interaction and those affecting ball-surface interaction. Regarding the later, a test method to assess the vertical ball rebound is included in both the CEN (European standard EN 12235) and the FIFA requirements (FIFA Test Method 01) [17]. In both cases, the test consists in a football falling vertically on the surface from a height of 2.00 ± 0.01 m (measured from the bottom of ball) without imparting any impulse or spin. After the ball hits the ground, the height to which it bounces is estimated by acoustic methods, and the percentage that the rebound height represents with respect to the initial dropping height is calculated.

Besides the complexity of the test due to the configuration and assembly of its components, which makes it one of the most time consuming and labor intensive tests for football pitches, this ball rebound test also lacks precision, since it estimates the rebound height from acoustic signals and this can induce errors, especially in windy conditions and noisy environments. To overcome this problem, a new device to evaluate the ball rebound has been developed. The objective of this study was to establish the accuracy of the new apparatus and compare it with that of the traditional method. The new test should facilitate the evaluation of the ball rebound in artificial turf football pitches, contributing to that the managing bodies of these facilities carry out a more systematic and exhaustive control of the functionality of the surface, decreasing notoriously the time for turf evaluation.

## 2. Novel Methodology

### 2.1. Standard Test Method

The principle of the test consists on releasing a ball from 2 m and determining the height of its first rebound from the surface.

For height estimation, an acoustic sensor records the time between the first and the second impact.

According to [17], for each test the rebound height can be determined by:

(1) $$h_{r}=1.23{\left(T-\Delta t\right)}^{2}\times 100$$

being $h_{r}$ the rebound height, *T* the time between first and second impact and Δt=0.025 s. The output value of ball rebound must be rounded to the nearest 0.01 m, since the allowed uncertainty of measurement is ±0.03 m.

The actual requirements of the device for ensuring a proper measurement are [17,19]:

- An electromagnetic or vacuum release mechanism that allows the ball to fall vertically from 2.00 ± 0.01 m (measured from the bottom of ball) without imparting any impulse or spin.
- Vertical scale to allow the drop height of the ball to be established.
- Timing device, activated acoustically, capable of measuring to an accuracy of 1 ms.
- Means of measuring wind speed to an accuracy of 0.1 m/s (field tests only).

**Figure 1:**
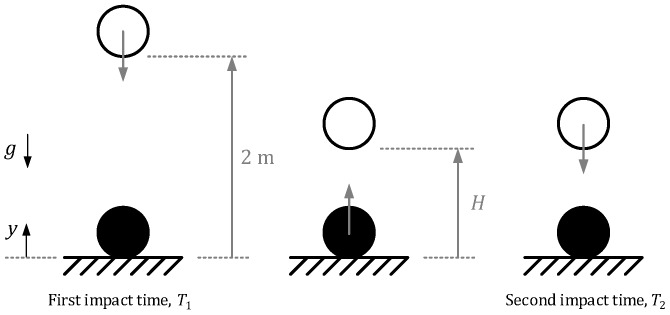
Conventional test method scenario.

Figure 1 illustrates the sequence of events of the conventional test method, where time between first and second impact is $T=T_{2}-T_{1}$.

### 2.2. Novel Proposal

The main disadvantages of the conventional test method are [17,19]:

- Slight initial spin is exerted and the rebound is not vertical.
- Acoustic measurement uses to yield incorrect measurement owing to any acoustic disturbance.
- Moderate wind presence yields to non-uniform rebound or acoustic disturbance and annuls the test.

Assuming a point mass model, the differential equation of the conventional test method is:

(2) $$m\ddot{y}\left(t\right)+c\dot{y}\left(t\right)=mg$$

being y(t) the vertical component of the ball movement, *m* the mass of the ball, *c* the viscous friction coefficient and *g* the gravity acceleration. For reproducing the dynamics of the test method the differential Equation (2) must be solved with the initial conditions y(0)=2−D/2 m, being *D* the diameter of the ball, and $\dot{y}\left(0\right)=0$ m/s. The ball rebound height corresponds to the maximum value of y(t) after the first rebound.

**Figure 2:**
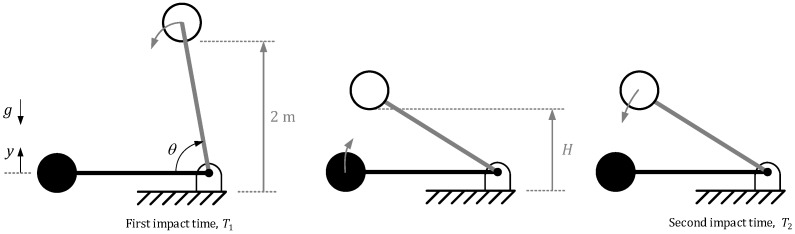
Proposed test method scenario.

In order to improve the conventional test method, a more direct estimation of the height of the rebound avoiding disturbances would be desirable. This proposal is based on modifying the free fall movement by a restricted movement as shown in Figure 2.

Assuming a point mass model, the differential equation of the proposed test method is:

(3) $$ml^{2}\ddot{\theta }\left(t\right)+c\dot{\theta }\left(t\right)=mg\cos\left(\theta (t)\right)l$$

where θ(t) is the beam angle with regards to horizontal plane. In this sense, the rebound height can be directly determined by measuring θ(t) and:

(4) y(t)=l·sin(θ(t))

Differential Equation (3) should be solved imposing a initial condition $\theta \left(0\right)=\theta_{0}$ rad and $\dot{\theta }\left(0\right)=0$ rad/s such as $l\sin(\theta_{0})=2$ m.

The rebound height ca be therefore obtained by means of:

(5) $$h_{r}=l\cdot \sin\left(\theta_{max}\right)$$

being $\theta_{max}$ the maximum angle measured by the encoder placed at the revolute joint after the first rebound.

This proposal avoids non-desired initial spin, non-uniform rebounds and it does not depend of any acoustic signal to measure the time between rebounds, and can directly obtain the rebound height by measuring angle θ, for example with an encoder.

Time constant of both dynamic models, (2) and (3), are different and the time that the ball takes in executing the trajectory is also different.

Nevertheless, for both models, the potential energy converts to kinetic energy and the rebound height should be similar.

On the other hand, the rebound is modeled by means for the coefficient of restitution (COR), assuming that the kinetic energy before and after the rebound is related by means of the COR, and:

(6) $$e=\frac{\dot{y}\left(t_{i}^{-}\right)}{\dot{y}\left(t_{i}^{+}\right)}$$

being $t_{i}^{-}$ the time before rebound and $t_{i}^{+}$ the time after it.

The following section demonstrates the analogous behavior, in term of rebound height, of scenarios shown in Figure 1 and Figure 2 by means of simulations.

## 3. Simulated Results

### 3.1. Preliminaries

In this section we present the dynamic simulations of both models. The simulations have been carried out using Simulink${}^{TM}$ of Matlab${}^{TM}$ software (R2019b, MathWorks, Massachusetts, United States). Sample time has been fixed to 1 ms.

The collision between turf and ball (Adidas conext 19, Adidas, Herzogenaurach, Germany) has been modeled using a illustrative coefficient of restitution of 0.825 (concrete) has been set and compared to the coefficient of restitution of artificial turf, 0.65 [20], but the analysis presented here is valid for any other values.

### 3.2. Comparative Results

**Figure 3:**
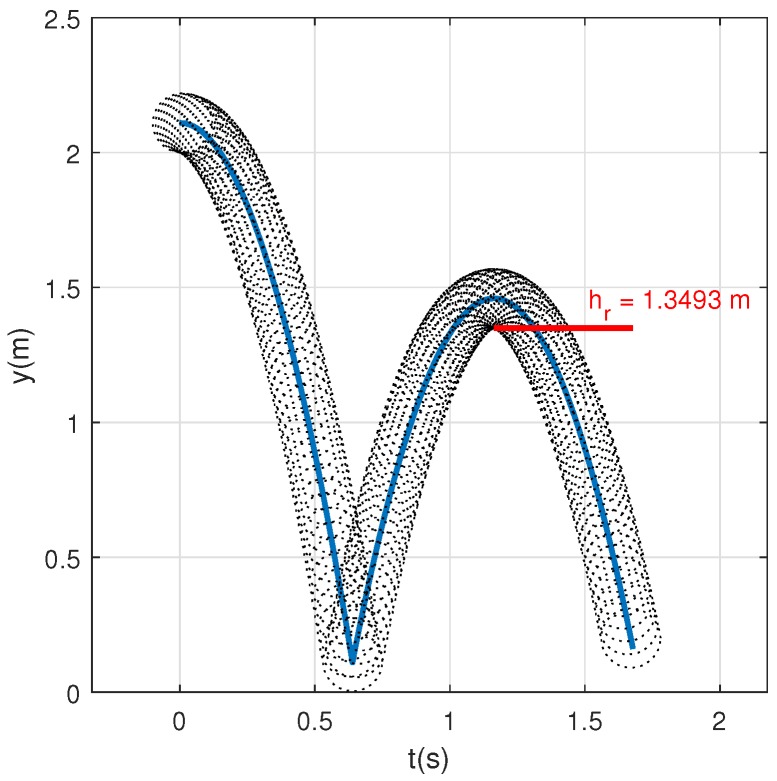
Conventional test method simulation.

Table 1 summaries the parameters used for the conventional test method since Figure 3 represents the evolution of the vertical coordinate of the ball during the test.

As reported in [17], the vertical ball rebound height should be 1.35 m when ball rebound on concrete. In this sense, for adjusting the COR value of the surface, a range of COR∈[0.2,1] has been simulated, choosing the COR value which provides the nearest value of rebound height to 1.35 m. The chosen value of COR is 0.825 which provides a value of rebound height 1.3493 m. (≈1.35 m).

In analogous way, Table 2 summaries the parameters used for the test method proposed here and Figure 3 represents the evolution of the angle of the beam, to be measured, and the vertical coordinate of the ball during the test.

**Figure 4:**
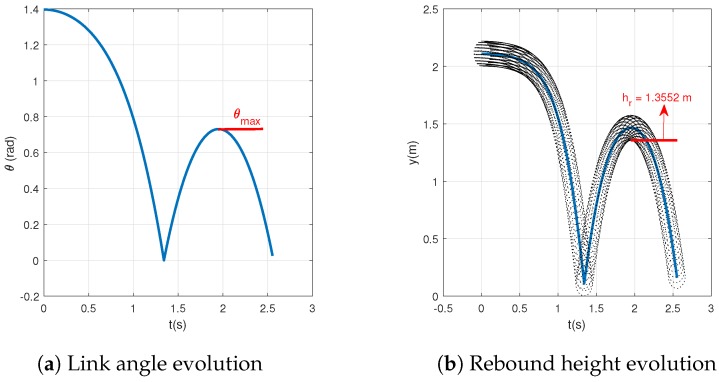
Proposed test method simulation.

Note that using the same COR (0.825) the obtained vertical ball is 1.3552 m, very close to the obtained with the conventional method (Figure 4).

The difference between both methods is the time that ball takes to rebound but the rebound maintains constant.

**Figure 5:**
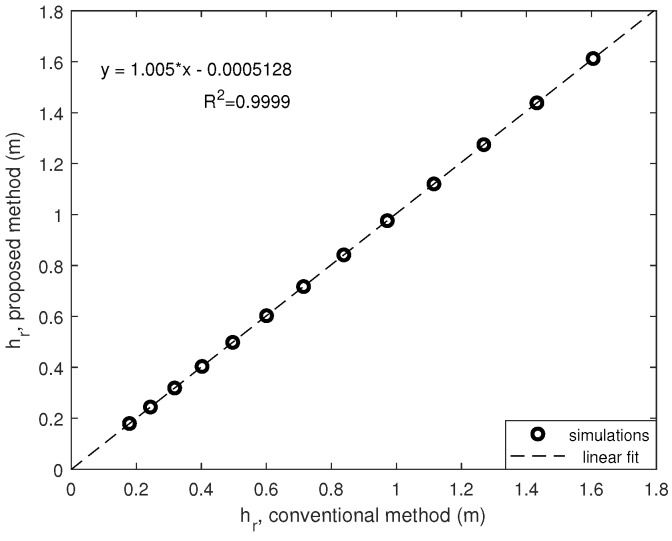
Ball rebound for different COR values: conventional vs. proposed.

Figure 5 compares the aforementioned simulations (conventional vs. proposal) by changing the COR in a reasonable range [0.3, 0.9]. Note that the linear fit is almost perfect, with a coefficient of correlation near to 1, and height of ball rebound are similar for both methods. Matlab${}^{TM}$ software Curve Fitting Tool (R2019b, MathWorks, Massachusetts, United States) was used to obtain the statistical data.

This result allows designing a device based in this proposal with the main advantage that the measurement of the height of the ball rebound does not require any acoustic sensor and a conventional encoder, placed at the joint of the beam, can be used for a directly measurement.

Finally, the simulated results have been compared to the experiments for validation purpose (Section 4.2). The comparison yields that model (3) is valid for describing the dynamics of the device (detailed in Section 4.1).

### 3.3. Scaling the Problem

In order to develop a more compact device based on this proposal, an analysis of the implications of scaling the problem is required.

**Figure 6:**
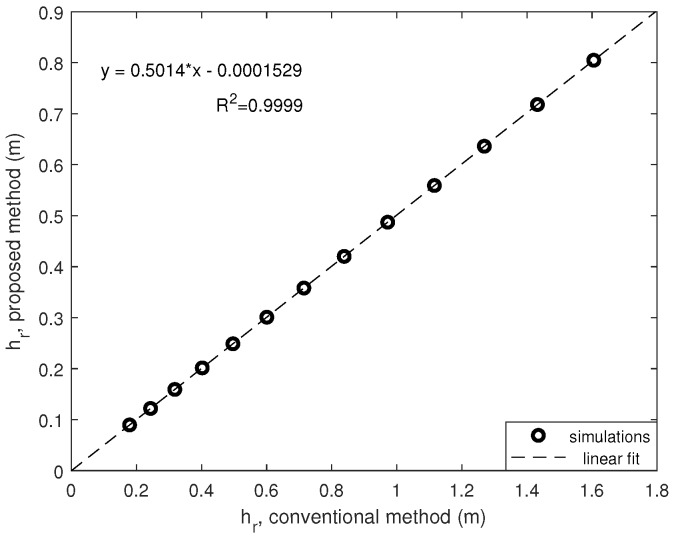
Ball rebound for different COR values: conventional vs. proposed.

Let’s assume that for the proposed method the length of the beam, $l^{*}$, is the half of the original one, i.e., 1 m. If we replicate the previous result by changing the length of the beam to 1 meter the results are represented in Figure 6.

Note that the goodness of the linear fit is almost perfect ($R^{2}\approx 1$) and the expected height of ball rebound is the half of the conventional one when decreasing the length of the beam to the half of the equivalent one.

In this sense, simulation results show that a compact device for ball rebound test method can be designed based in this proposal and the height of the rebound can be obtained as:

(7) $$h_{r}=\frac{2}{l^{*}}\cdot h_{r}^{l^{*}}$$

being $h_{r}^{l^{*}}$ the rebound height obtained using $l^{*}$ as beam length.

## 4. Experimental Results

### 4.1. Prototype and Height of Ball Rebound

**Figure 7:**
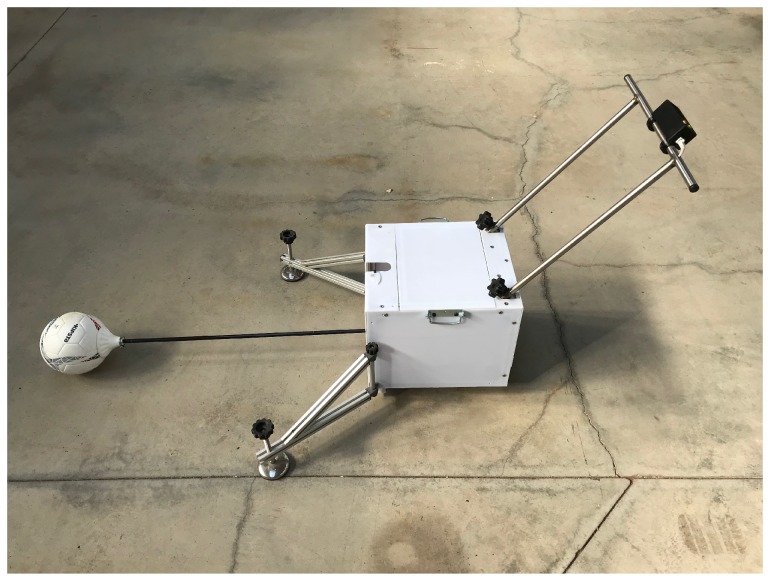
First prototype.

The first prototype for testing this proposal is shown in Figure 7.

The prototype has an aluminum frame covered by a polycarbonate case. Inside the frame, a motor/gearbox set lifts the set beam/ball up to 80${}^{\circ }$ and allows the beam to fall down. An incremental encoder obtains the angular position of the beam and it is registered in a microcontroller.

The length of the beam is 1 m for compacting purpose and the equivalent height of the rebound can be therefore obtained as:

(8) $$h_{r}=C\cdot h_{r}^{1}=C\cdot \sin\left(\theta_{max}\right)$$

being *C* an invariant coefficient that should be experimentally determined, $h_{r}^{1}$ the height of the ball rebound of the novel device and $\theta_{max}$ the maximum angle measured by the encoder after the first rebound.

The beam is a 10 mm of diameter carbon fiber tube and the ball is attached to the tip by means of a part printed in PLA (polylactic acid).

### 4.2. Device Validation

For determining vertical rebound height is only required to obtain the maximum angle after the first ball rebound ($\theta_{max}$). Nevertheless, in order to validate the dynamics model (3) a set of experiments obtaining the whole ball trajectory (movement / impact / rebound movement) has been developed.

Controller has been configured to determine the angle of the ball, θ(t), during all the trajectory using a sample time of 10 ms.

**Figure 8:**
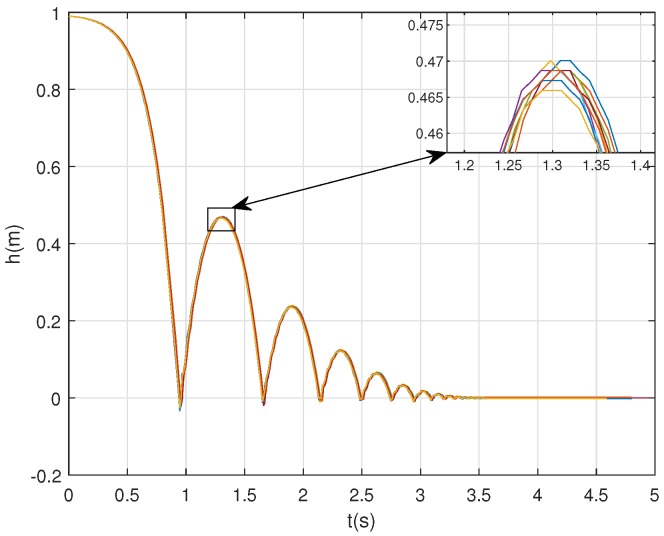
Experimental results: trajectory of the ball. Set of 10 tests for model validation purpose.

In order to demonstrate the repeatability of the device, not only in the final value of the rebound height (see Section 4.4), but also the trajectory of the ball, Figure 8 represents the trajectory of the ball for a set of 10 experiments in a concrete surface. Note that all the trajectories described by the ball during the 10 different test are similar. The maximum error committed between the 10 test is 0.87cm.

Once that we have demonstrated that the device provides repeatable trajectories of the ball, we are going to compare the experimental results with the mathematical model (3).

**Figure 9:**
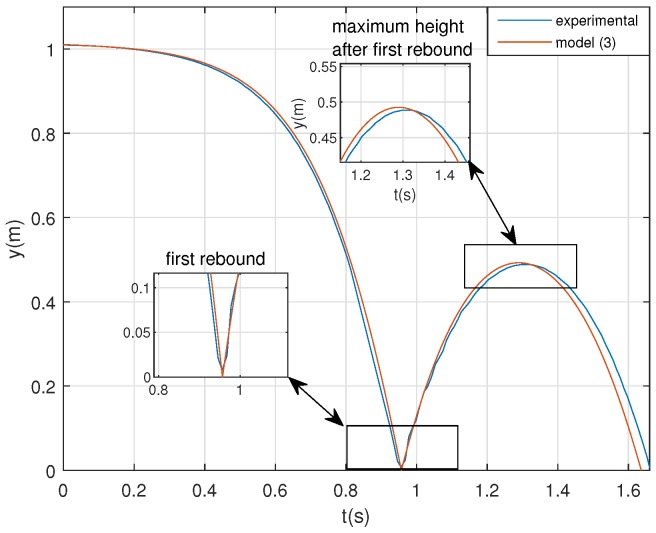
Simulation vs. experimental result.

Using the parameters shown in Table 3, Figure 9 compares simulation result to the experimental one (e.g., test 5).

Note that, although some time delay between both results is noticeable after the first rebound, model (3) describes the dynamics of the real prototype. In this sense, the results demonstrate that model (3) is accurate enough (more than a fit of 80%, see e.g., [21]) for describing the dynamics of the system. Other effects such as non-linear friction, complex impact models or aerodynamics force, are not needed and simple model (3) is enough to describe the dynamics of the device.

### 4.3. Calibration Test

The first set of experiments allows to obtain the linear coefficient which relates the height of the novel device with regards to the conventional one. Attending to [17], a initial test on concrete should be carried out to adjust the pressure of the ball for a rebound height of 1.35 m with the conventional device. For this calibration a set of 20 experiments on concrete has been carried out with both devices. The obtained results are summarized in Table 4.

Using the Method of Least Squares the value of coefficient *C* of [8] can be easily obtained and yields, C=2.8435. In this sense, the calibration equation for obtaining the ball rebound test results for this particular device is:

(9) $$h_{r}=2.8435\cdot \sin\left(\theta_{max}\right)$$

### 4.4. Repeatability and Accuracy

After the calibration procedure, a set of experiments to check the repeatability of the results is required. For this purpose, 3 different surfaces have been using both, conventional and novel device. A set of 20 tests has been carried out for each surface and for each device.

**Figure 10:**
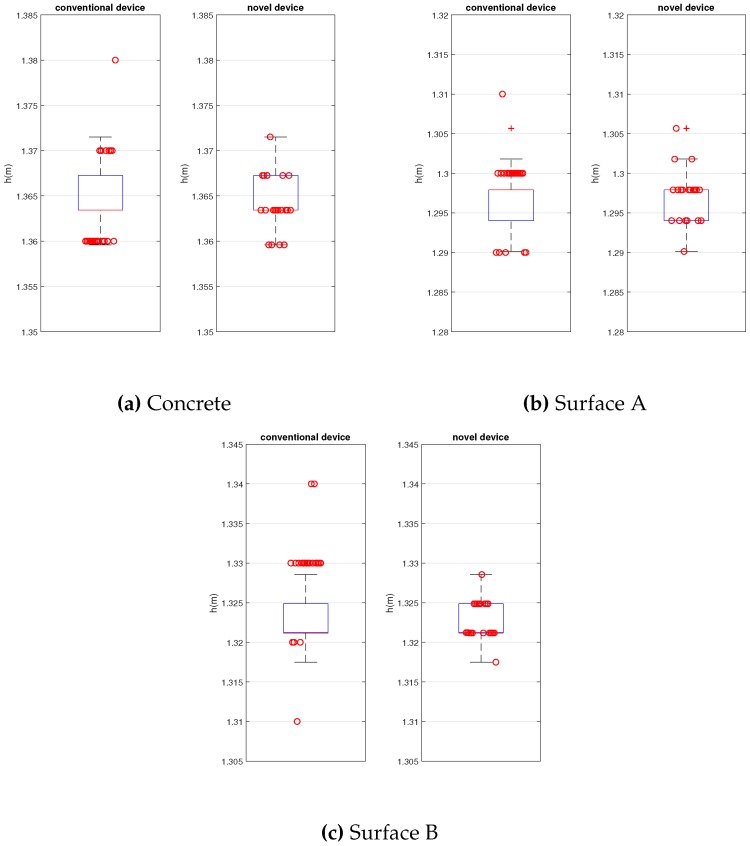
Repeatability and accuracy tests.

Figure 10 represents the Box Plot of the results of both devices for the 3 different surfaces. Matlab${}^{TM}$ software (R2019b, MathWorks, Massachusetts, United States) was used to graph.

Note that the results of both devices are very close in terms of value and dispersion. The proposed device results the same values of ball rebound that the conventional one and a bit lower dispersion.

### 4.5. Final Test: Turf Evaluation

**Figure 11:**
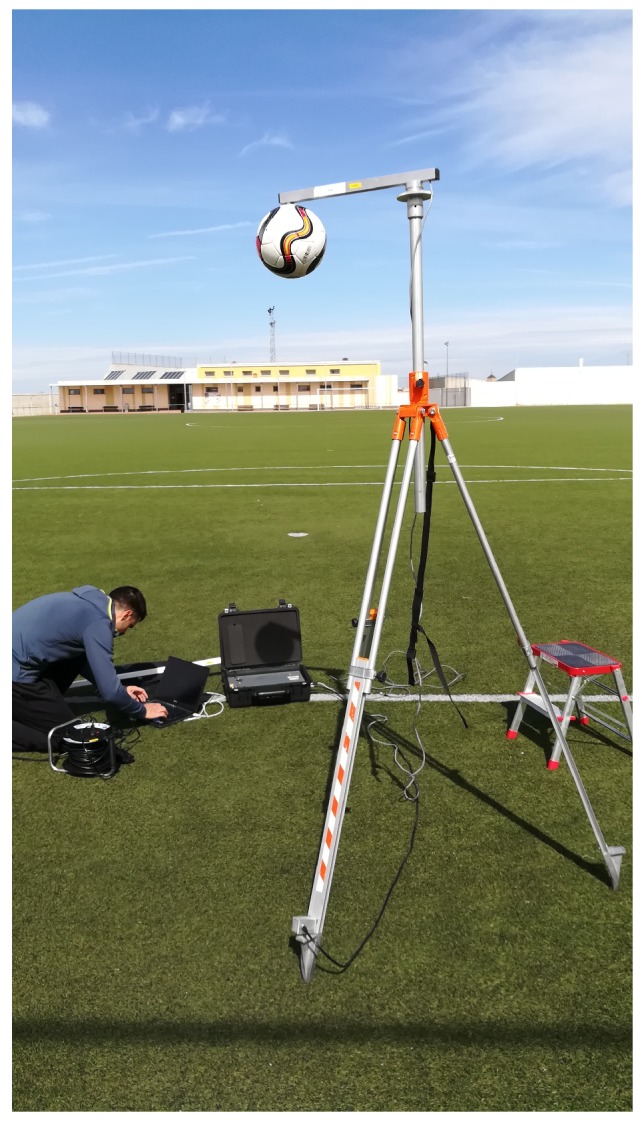
Certified device for determining football rebound.

For demonstrating the final application of the novel device for turf evaluation, a real artificial turf field has been tested with the conventional (certified device, see Figure 11) and novel device (see Figure 7) for comparison purpose. All the tests have been carried out following the Fifa Quality Programme procedure [17].

**Figure 12:**
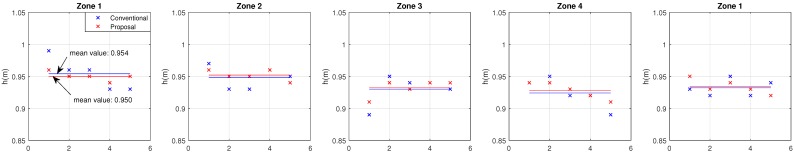
Compared evaluation: Villacanas field.

Table 5 and Figure 12 compares the obtained results of ball rebound test with both devices in the evaluation of Villacanas field (Toledo, Spain). Obtaining *p*-value between conventional and novel device results (Matlab${}^{TM}$ software (R2019b, MathWorks, MA, USA) we can conclude that both results are analogous and no noticeable difference can be found between them ($p=3.586\cdot 10^{-6}$). Table 5 also includes test time, wasting time of non-valid tests, moving the equipment between zones and equipment setup.

Owing to the preparation procedure of the conventional test and the acoustic based measurement, the effective time for evaluating the field with the conventional device (see Figure 11) was 65.2 min, since the time with the new device was about 8.30 min.

## 5. Conclusions

Assessing and keeping control of the mechanical properties of sport surfaces is a relevant task in sports since it enables athletes to train and compete safely and under equal conditions. This is particularly important in synthetic, complex surfaces whose properties evolve over time and usage, such as artificial turf pitches.

Currently, different tests are used for assessing athlete- and ball-surface interactions in artificial turf pitches. In order to make these evaluations more agile and accessible for every facility, it is important to develop new apparatus that enable to perform the tests in an easier and quicker way.

In this sense, the existing equipment for determining the vertical ball behavior requires a complex and non-easily transportable device (see Figure 1) in which the ball must be fixed to the upper part of the frame in a very precise position by means of a magnet. After release, the rebound height is determined by capturing the acoustic signal produced when the ball bounces on the turf. When extended tests are conducted, the time required to evaluate a single field is too high due to the non-valid trials (for undesired initial spin or acoustic interference) together with the waste of time that takes the user to gather the ball and fix it back to the magnet.

This work proposes a novel methodology which allows to notoriously decrease the time of testing fields maintaining the repeatability and accuracy of the test method together with a compact device for improving its mobility and transport (see Figure 7).

The proposal is based on fixing the ball to a very lightweight beam and reproducing the tests with a constraining rotational movement and estimating the rebound height with a conventional encoder placed in the rotational joint. By means of simulations we have demonstrated that the rebound height of both movements, the free and the rotational one, is the same and the problem can be also easily scalable.

A preliminary prototype has been built and tested based on this new proposal. This prototype has a motor/gearbox set which allows an automated placing of the ball at the beginning of the test and encoder to measure the angle of the beam during the test. The repeatability and the accuracy of the device have been experimentally tested over three surfaces.

Finally, a field has been evaluated with both devices for comparing purpose. The novel proposal maintains the quality of the evaluation but wastes much less time for the whole evaluation of the field. Additionally, the device can be used by a non-specialized user.

## Figures and Tables

**Table 1:** Simulation parameter of the conventional test method.

Parameter	Value
Mass of the ball	0.43 kg
Diameter of the ball	0.22 m
Coefficient of restitution	0.825 m
Coefficient of friction (air)	5·$10^{-3}$ m
Initial condition, y(0)	2.00 m

**Table 2:** Simulation parameter of the proposal test method.

Parameter	Value
Mass of the ball	0.43 kg
Diameter of the ball	0.22 m
Initial condition, θ(0)	80${}^{\circ }$
Beam length	2.0308 m

**Table 3:** Simulation parameter for model validation

Parameter	Value
Mass of the ball	0.54 kg
Diameter of the ball	0.22 m
Initial condition, θ(0)	82${}^{\circ }$
Beam length	1.01 m

**Table 4:** Calibration tests: concrete surface.

Test	Conventional	Novel	
	Device	Device	
**id.**	**h (m)**	**$\theta_{max}$ (${}^{\circ }$)**	**$\sin(\theta_{max})$**
1	1.36	28.652	0.4795
2	1.36	28.740	0.4808
3	1.36	28.740	0.4808
4	1.36	28.652	0.4795
5	1.36	28.740	0.4808
6	1.36	28.564	0.4781
7	1.36	28.838	0.4823
8	1.36	28.564	0.4781
9	1.36	28.652	0.4795
10	1.37	28.652	0.4795
11	1.37	28.652	0.4795
12	1.36	28.652	0.4795
13	1.36	28.564	0.4781
14	1.37	28.652	0.4795
15	1.36	28.740	0.4808
16	1.37	28.564	0.4781
17	1.37	28.652	0.4795
18	1.37	28.652	0.4795
19	1.36	28.740	0.4808
20	1.38	28.652	0.4795
minimum value	1.36	28.564	0.4781
median value	1.36	28.652	0.4795
maximum value	1.38	28.838	0.4823

**Table 5:** Compared evaluation: Villacanas field.

Field Zone	Conventional Device			Novel Device		
	**Value (m)**	**Test Time (s)**	**Mean (m)**	**Value (m)**	**Test Time (s)**	**Mean (m)**
Zone 1	0.99	32	0.954	0.96	5	0.950
	0.96	31		0.95	4	
	0.96	29		0.95	5	
	0.93	27		0.94	4	
	0.93	28		0.95	4	
Zone 2	0.97	30	0.948	0.96	4	0.952
	0.93	31		0.95	4	
	0.93	33		0.95	5	
	0.96	34		0.96	4	
	0.95	35		0.94	4	
Zone 3	0.89	37	0.930	0.91	4	0.932
	0.95	32		0.94	5	
	0.94	28		0.93	4	
	0.94	27		0.94	4	
	0.93	28		0.94	5	
Zone 4	0.94	33	0.924	0.94	5	0.928
	0.95	32		0.94	5	
	0.92	24		0.93	5	
	0.92	32		0.92	4	
	0.89	36		0.91	4	
Zone 5	0.93	34	0.932	0.95	4	0.934
	0.92	42		0.93	4	
	0.95	28		0.94	4	
	0.92	32		0.93	5	
	0.94	36		0.92		
Wasting time	Conventional device			Novel device		
Non-valid test (s)	854			0		
Equipment movement (s)	1035			265		
Equipment setup (s)	1232			123		
TOTAL EVALUATION TIME (min)	65.20			8.30		
